# A Lung Ultrasound Scanning Technique for Children and Adults in Low-Resource Settings: Preliminary Experiences in Sub-Saharan Africa

**DOI:** 10.4269/ajtmh.20-0272

**Published:** 2021-09-27

**Authors:** Matthew Fentress, Phillip Ezibon, Akuot Bulabek, Carla Schwanfelder, David Schrift, Sachita Shah, James Tsung, Adi Nadimpalli

**Affiliations:** ^1^ Medecins Sans Frontieres, Geneva, Switzerland;; ^2^ Medecins Sans Frontieres, Juba, South Sudan;; ^3^ Prisma Health University of South Carolina Medical Group, Division of Pulmonary, Critical Care and Sleep Medicine, Columbia, South Carolina;; ^4^ Department of Emergency Medicine, University of Washington, Seattle, Washington;; ^5^ Department of Emergency Medicine, Mount Sinai Medical Center, New York, New York;; ^6^ Medecins Sans Frontieres, New York, New York

## Abstract

Lung ultrasound is increasingly used as a diagnostic tool for pulmonary pathologies by nonspecialist clinicians in resource-limited settings where chest X-ray may not be readily available. However, the optimal technique for lung ultrasound in these settings is not yet clearly defined. We describe here our experience of implementing a standardized, focused six-zone, 12-view lung ultrasound scanning technique with a high-frequency probe in both adults and children in a resource-limited setting in sub-Saharan Africa. Our experience suggests that this may be a feasible technique to rapidly introduce lung ultrasound to new learners that can be adapted to emergency or outbreak settings. However, research is needed to determine how this technique compares with clinical examination and other available tests for the diagnosis of pathology commonly encountered in resource-limited settings.

## INTRODUCTION

**Figure 1.**
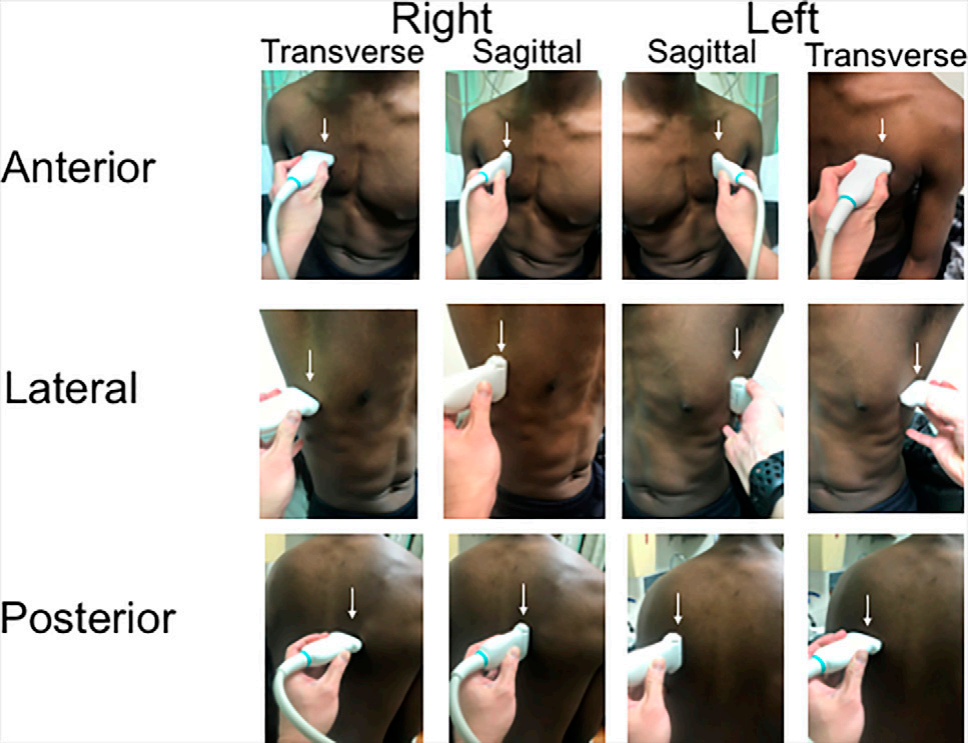
Six-zone, 12-view scanning protocol. The technique involves sliding from the apex to diaphragm in six zones—anterior (midclavicular line), lateral (midaxillary line), and posterior (paraspinal line) bilaterally—in both sagittal and transverse orientations. See Supplemental Appendix 1 for labeling nomenclature. This figure appears in color at www.ajtmh.org.

Lung ultrasound (LUS) is increasingly used as a diagnostic modality for respiratory pathologies. It can be rapidly performed at the patient’s bedside by the treating clinician and provide real-time information, while minimizing the radiation harm and delay associated with X-rays. International consensus recommendations have helped to standardize the methods, image interpretation, and related terminology of LUS^1^ to diagnose lung consolidations, alveolar interstitial syndromes, and pleural effusions. Widely used techniques for adults use low-frequency probes (either curvilinear or phased-array probes), which are useful for identifying deeper structures. Examples of these techniques include those described by Volpicelli^2^ and Lichtenstein,^3^ which involve placing the probe in six or eight zones of the anterolateral chest, and a more comprehensive protocol described by Reissig,^4^ which involves systematic interrogation of each rib space. For pediatric patients, Copetti^5^ showed that LUS can be as accurate as chest X-ray (CXR) for the diagnosis of pneumonia, using both the low-frequency convex and high-frequency linear probes. The high-frequency linear probes provide better image quality and resolution at shallower depths, which is advantageous in pediatric patients who have smaller body habitus. Tsung et al.^6^ modified these pediatric techniques by exclusively using a high-frequency linear probe for pediatric patients, sliding from the top of the chest to the bottom in both the longitudinal and transverse planes, resulting in a six-zone, 12-view protocol (Figure 1). This technique has the added advantage of quickly covering most of the anterior, lateral, and posterior aspects of both lungs, which is necessary to optimize diagnostic yield when pathology may be limited to a single lobe or lung. In clinical practice, however, the choice of technique normally depends on sonologist preference, probe availability, age, and body habitus of the patient.

The potential benefits of using LUS for respiratory pathologies are amplified in low-resource settings, where the burden of respiratory diseases is often higher. The miniaturization of ultrasound machines and cost reductions may make them more accessible than X-ray machines. Unfortunately, implementing LUS in low-resource settings remains challenging. First, ultrasound image quality depends on qualified sonographers, who may be lacking in many sites. Second, there is scant literature to guide which of the LUS techniques just described is optimal for patients in different contexts. Although different techniques have their benefits, LUS is fundamentally an analysis of the artifacts that different pathologies produce on the ultrasound monitor.^1^ Thus, a key challenge is to standardize image acquisition techniques so that the clinician can analyze the potential artifacts for accurate disease diagnosis. Third, whichever technique is chosen, it must be flexible enough to cover both adults and children because clinicians in low-resource settings routinely care for both populations. The ideal technique should be simple, standardized, scan both lungs thoroughly, and preferably use just one probe because a frequent solution to the problem of physician deficits has been to “task-shift” clinical responsibilities to midlevel providers and nurses through protocolization.

To help inform others considering use of LUS in a resource-limited setting, we present our experience in implementing LUS for adult and pediatric patients through longitudinal training of both midlevel providers (clinical officers, COs) and physicians in Agok, South Sudan, using a single high-frequency probe technique for all patients.

## SETTING AND TECHNIQUE

In 2017, we demonstrated the feasibility of a short training program for COs for pediatric LUS using the single linear probe methodology by Tsung in the pediatric department of the Aweil General Hospital, in Aweil, South Sudan.^7^ Subsequently, in 2018, we embarked on implementing LUS in a separate Médecins Sans Frontières (MSF)-run hospital in Agok, South Sudan. This was a 140-bed rural hospital in Abyei Special Administrative Area, the only referral hospital in the region, with a catchment area of more than 140,000 and service to patients of all ages. These clinicians routinely care for both adult and pediatric patients, which differed from the experiences of teaching pediatric LUS in Aweil. The majority of patient care decisions were made by COs supervised by a few physicians. Consequently, our main challenge was to develop a longitudinal training program for novice, nonphysician trainees and to standardize the LUS technique that was harmonized across both pediatric and adult patients. Our solutions followed other precedents of adapting LUS applications to resource-limited settings.^7^^,^^8^ Of note, at the time of LUS introduction in Agok, no other diagnostic imaging was available apart from ultrasound, although an X-ray machine was installed approximately 1 year later. The LUS implementation was considered a quality improvement project, and the data presented here are routine programmatic data; therefore, formal ethics approval was not required. Written informed consent was obtained from the patients whose ultrasound images are included.

To harmonize the LUS technique across adult and pediatric patients, we deliberated with both expert LUS sonologists and the Agok Hospital medical management team. The simplest proposed solution was to use a single methodology for both the adult and pediatric patients. The indication for performing an ultrasound was any type of respiratory distress or chest pain for any patients, after a physical examination and history-taking. Given the difficulty in using the low-frequency probes in children, who have shallower pleurae, we settled on using a single high-frequency linear probe methodology with a six-zone, 12-view scanning technique, as originally described for pediatrics by Copetti^5^ and adapted by Tsung,^6^ for both pediatric and adult patients. The technique involved sliding from apices to diaphragm in six zones (anterior, lateral, and posterior bilaterally) in sagittal and transverse orientations (Figure 1). Initial depth was set at 3.5 cm, and trainees were advised to adjust depth as necessary so that at least one A line was visible in the far field. Clips of the entire sweep from each of the 12 views were saved with appropriate labeling for documentation (Supplemental Appendix 1) and could be reviewed by an expert reviewer/specialist if needed for diagnostic assistance or quality assurance. A Sonosite M-Turbo (Bothell, WA) with 13-6 MHz probe and Philips Lumify (Amsterdam, the Netherlands) with a 12-4 MHz probe were used for all imaging.

## EXPERIENCES

For the training, we used a field-based, “probe-in-hand” model that emphasized scanning and image review over formal didactic sessions. Trainees were selected by a competitive application process from a group of 19 clinicians, including 14 COs and five MDs. Six COs and four physicians were in the initial cadre of trainees, with the trainer being a physician with expertise in both LUS and other point-of-care Ultrasound (POCUS). Two members of this trainee group had introductory-level experience in nonpulmonary ultrasound, one CO and one MD, and the remainder were ultrasound naive. Over a month-long timespan, each trainee had three to four dedicated days of LUS training. Each training day had 1 hour of didactics, followed by 7 hours of ultrasound scanning and image review with the trainer (Supplemental Appendix 2). Scanning was performed at the bedside on patients with both normal and abnormal lung findings. There were between two to three trainees per day, and each trainee had access to one ultrasound machine for the entire day. A month-long refresher training was provided 9 months after the initial training.

Trainees were qualified to perform LUS independently when they submitted 25 LUS exams that were validated by the trainer through a formal, on-site evaluation process incorporated into the initial and refresher trainings. Four ultrasound machines were available during the trainings, and two machines during nontraining time. Local guidelines were developed for use of LUS versus chest radiography, and indications evolved depending on a number of factors, including the functioning of the X-ray machine and infection prevention and control. Daily device care was the responsibility of the user, and routine maintenance reviews were conducted by an on-site biomedical technician every 3 months, or more frequently if a problem arose. Quality assurance for this implementation was performed in a semirandom fashion by telemedicine consultation, review by local POCUS focal points (one MD and one CO), and expert trainers during on-site trainings.

The first training was held in Agok in February 2018, followed by the refresher training in November 2018. During these trainings, six COs and four physicians were trained and validated in LUS. These clinicians then performed LUS independently as part of their clinical responsibilities, and integrated LUS findings into their clinical decision making. Between March and December 2018 the clinicians collectively performed 211 LUS exams, and between January and November 2019, they performed 183, for a total of 394 exams during the study period. Clinical officers performed 60% (236) of the exams, and MDs performed 40% (158). Of the 183 exams in 2019, 81 (44%) were done on children <15 years old, and 102 (56%) were done in adults >15 years old. Unfortunately, age distribution was not recorded in 2018.

Overall, on the basis of the experience of field-based clinicians, visiting trainers, and a selection of cases submitted to the internal MSF Telemedicine service for expert radiology review, our impression is that the trainees were able to perform LUS adequately and that the single high-frequency probe method was generally acceptable for interpretation of the common pathologic conditions in both adult and pediatric patients. In this setting, the main pathologies seen on LUS were lung consolidation, small subpleural consolidations, pleural effusion, and, less commonly, interstitial syndrome (e.g., pulmonary edema). Infrequently, the low-frequency probe was necessary to resolve uncertain pulmonary findings such as large pleural effusions or deep consolidations, particularly in cases of increased adiposity or muscle mass, although we did not record the exact number of times this was done.

**Figure 2.**
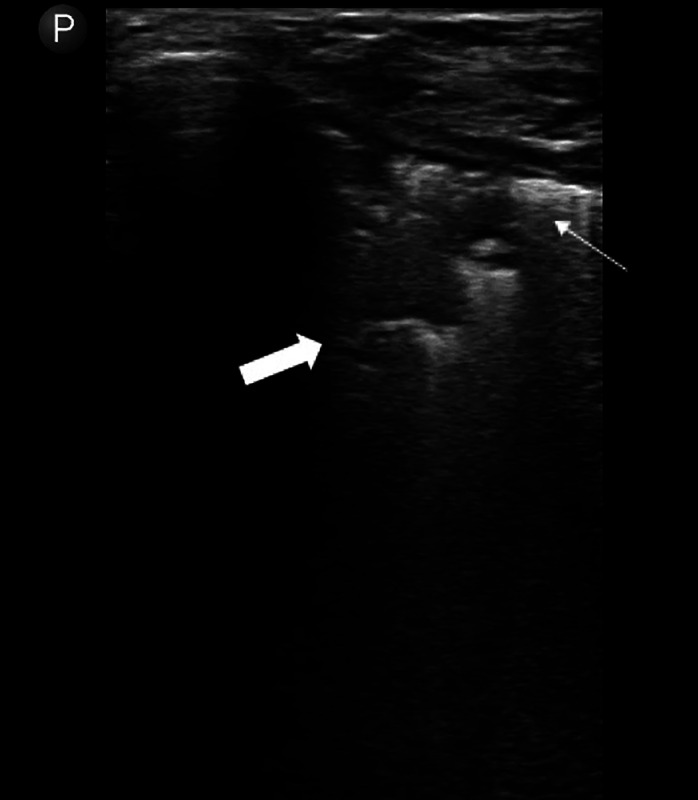
Apical consolidation (thick arrow) adjacent to pleural line (thin arrow) in 30-year-old patient with 3 months productive cough, fever, and weakness. Sputum smear was positive for acid-fast bacillus. Lung ultrasound demonstrated bilateral upper lobe consolidations and absent lung sliding in bilateral anterior fields. The patient was treated for pulmonary tuberculosis.

**Figure 3.**
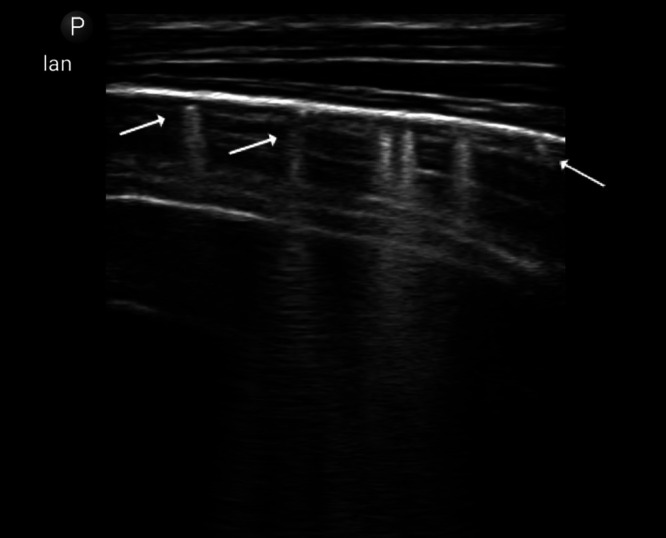
Sonographic miliary pattern, characterized by B-lines (arrowhead) and subpleural granularity (thin arrows) in almost every scanning zone, best seen during respiratory movement (Supplementary Video 1). This 40-year-old HIV-positive patient presented with 2 months of fever, cough, and weight loss and was unable to produce sputum for acid-fast bacillus testing. Abdominal and cardiac imaging showed splenic microabscesses and a small pericardial effusion, further supporting a diagnosis of disseminated tuberculosis. The patient was started on treatment for tuberculosis and adult malnutrition and discharged home with gradual improvement after 10 days.

Two select cases using this technique in adults are described in Figures 2 and 3.

## DISCUSSION

We describe here our experience implementing a single LUS technique for both adults and children in a low-resource setting in South Sudan. The implementation consisted of training novice users with a model that emphasized practical skills and providing sufficient ultrasound machines for the trainees to complete a minimum number of studies. Given the challenges of training novice users and the time constraints, we chose to merge different LUS techniques into a standard six-zone, 12-view protocol performed with a high-frequency probe for both adults and pediatrics. To our knowledge, this unifying technique has not been widely described in resource-limited settings. Our current experience suggests that this LUS technique may be a feasible model to expand a novel and important diagnostic modality for both adults and children in low-resource settings. In addition, the simplicity and universality of this model allow scaling up LUS in emergencies and outbreak settings.

Although our study period predated the COVID-19 pandemic, numerous studies published since January 2020 have reported on the potential use of LUS for screening and diagnosis of COVID pneumonia.^9^^–^^11^ The technique described here can be used to detect lesions in COVID pneumonia and is used for this purpose in several MSF projects.

### Benefits and limitations.

The primary benefit of this technique is that it can be taught with relative ease to nonphysician clinicians as a single technique for both pediatrics and adults. Especially in field-based trainings with time constraints and limited ultrasound machines, using a single technique is markedly simpler to implement. Furthermore, use of the high-frequency probe provides excellent image clarity and high resolution for various infectious pathology, specifically lung consolidations and pleural effusions. In our experience, this technique may also increase diagnostic yield relative to methods scanning only the anterolateral chest in certain populations, including those who may have tuberculosis (apical lesions) or ambulatory patients with pneumonia (possible posterior lung consolidations). The technique is also less time-consuming than a systematic exam of each intercostal space described in the literature, however not completing the entire scanning protocol also risks missed pneumonia.^12^ The most important limitation of the technique is that there are limited data on its utility in adults, although it appears to have adequate accuracy in children and young adults.^13^ It may also be more difficult to obtain adequate images in patients with a large body habitus using a linear transducer, it carries the potential for underdiagnosis given the narrow field of view of the linear probe, and it may require the use of a lower frequency curvilinear probe to fully visualize large pleural effusions or deep consolidations.

### Future steps.

Prospective research should be conducted to determine how this technique compares with other published techniques for the diagnosis of pathologies commonly encountered in this context, especially pulmonary tuberculosis. Given its relative simplicity and reproducibility, if it compares favorably to other scanning protocols, it has the potential to be more widely applied in resource-limited settings with nonspecialized clinicians.

## CONCLUSION

A standardized six-zone, 12-view LUS technique using a high-frequency probe for both adults and children may be a feasible technique to rapidly introduce LUS to new learners in resource-limited settings. Further research is needed to determine how this technique performs in comparison with other published techniques for diagnosis of various lung pathologies, especially those commonly encountered in resource-limited settings.

## Supplemental Material


Supplemental materials
