# Supplementary material for: A Lung Ultrasound Scanning Technique for Children and Adults in Low-Resource Settings: Preliminary Experiences in Sub-Saharan Africa
Source: Am J Trop Med Hyg. 2021 Sep 27;105(5):1148–51. doi: 10.4269/ajtmh.20-0272 (PMC8592227; doi:10.4269/ajtmh.20-0272)
Supplement: Supplementary file 1 [file tpmd200272.SD1.pdf]

Supplemental Appendix 1 – Labeling nomenclature as detailed to the trainees

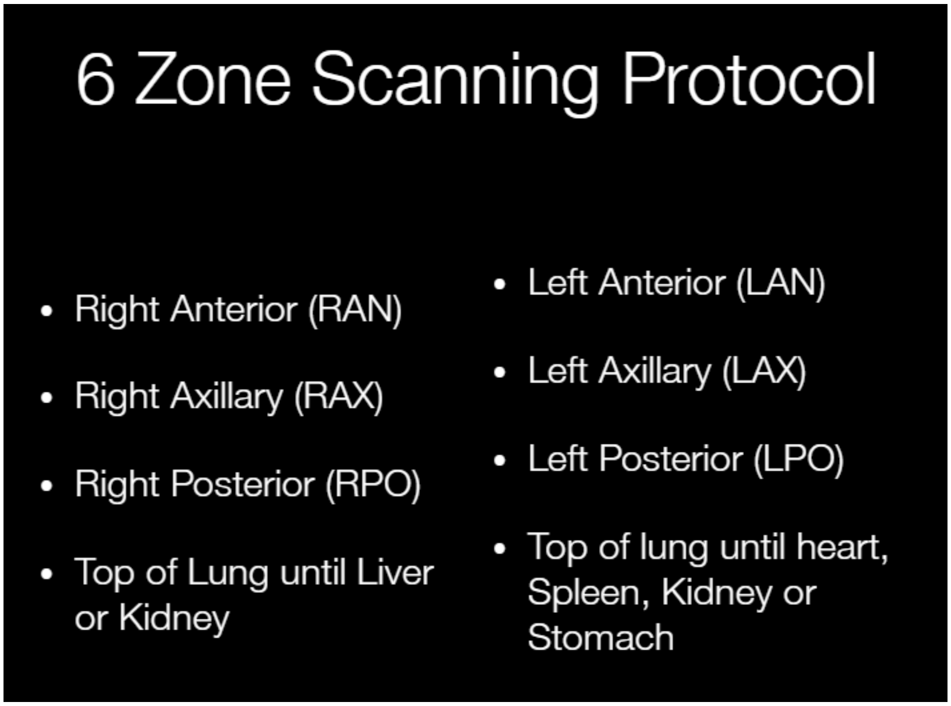

Supplemental Appendix 2 – Training Methodology

We developed the 'field-based probe-in-hand' training method by adapting from other trainings described in the medical literature. This model emphasizes ultrasound scanning time and integrated clinical decision making. Simultaneously, it de-emphasizes unnecessary theoretical aspects of ultrasound physics- and limited didactic lectures in general – which are frequently over-emphasized in other POCUS courses. We designed the implementation to add an extra tool to the clinician's diagnostic arsenal, along with the history, physical and rapid tests, rather than seek to create expert radiologists.

A standardized roster for 6 trainees can be made over a 4-week period, which gives each participant 8 days (56 hours) of dedicated training time. A typical daily agenda for POCUS implementation, involving one trainer and two learners per day, is shown below in Table 1.

Table 1: Daily Agenda for POCUS implementation

Time	Agenda
8-9am	1 hour – didactic lecture - trainer teaches through PowerPoint and image review
9-10am	1 hour – bedside - trainer helps learners acquire proper ultrasound images at bedside
10am-12:30pm	2.5 hours – bedside - learners acquire images on own & complete clinical pathway sheets
12:30-1pm	0.5 hour – ward - trainer and learners review morning images and clinical pathway sheets
1-2pm	Lunch break

2-2:30pm	0.5 hour - ward – 10 min didactic, 20 min bedside training
2:30-4:30pm	2hrs - bedside - learners acquire images on own & complete clinical pathway sheets
4-5pm	1 hour – Ward - review images/ clinical pathway sheets, final didactic and review
